# Early Target Cells of Measles Virus after Aerosol Infection of Non-Human Primates

**DOI:** 10.1371/journal.ppat.1001263

**Published:** 2011-01-27

**Authors:** Ken Lemon, Rory D. de Vries, Annelies W. Mesman, Stephen McQuaid, Geert van Amerongen, Selma Yüksel, Martin Ludlow, Linda J. Rennick, Thijs Kuiken, Bertus K. Rima, Teunis B. H. Geijtenbeek, Albert D. M. E. Osterhaus, W. Paul Duprex, Rik L. de Swart

**Affiliations:** 1 Centre for Infection and Immunity, School of Medicine, Dentistry and Biomedical Sciences, Queen's University of Belfast, Belfast, United Kingdom; 2 Department of Virology, Erasmus MC, Rotterdam, The Netherlands; 3 Centre for Experimental and Molecular Medicine, Academic Medical Center, Amsterdam, The Netherlands; 4 Tissue Pathology, Belfast Health and Social Care Trust, Queen's University of Belfast, Belfast, United Kingdom; 5 Department of Microbiology, Boston University School of Medicine, Boston, Massachusetts, United States of America; Dalhousie University, Canada

## Abstract

Measles virus (MV) is highly infectious, and has long been thought to enter the host by infecting epithelial cells of the respiratory tract. However, epithelial cells do not express signaling lymphocyte activation molecule (CD150), which is the high-affinity cellular receptor for wild-type MV strains. We have generated a new recombinant MV strain expressing enhanced green fluorescent protein (EGFP), based on a wild-type genotype B3 virus isolate from Khartoum, Sudan (KS). Cynomolgus macaques were infected with a high dose of rMV^KS^EGFP by aerosol inhalation to ensure that the virus could reach the full range of potential target cells throughout the entire respiratory tract. Animals were euthanized 2, 3, 4 or 5 days post-infection (d.p.i., n = 3 per time point) and infected (EGFP^+^) cells were identified at all four time points, albeit at low levels 2 and 3 d.p.i. At these earliest time points, MV-infected cells were exclusively detected in the lungs by fluorescence microscopy, histopathology and/or virus isolation from broncho-alveolar lavage cells. On 2 d.p.i., EGFP^+^ cells were phenotypically typed as large mononuclear cells present in the alveolar lumen or lining the alveolar epithelium. One to two days later, larger clusters of MV-infected cells were detected in bronchus-associated lymphoid tissue (BALT) and in the tracheo-bronchial lymph nodes. From 4 d.p.i. onward, MV-infected cells were detected in peripheral blood and various lymphoid tissues. In spite of the possibility for the aerosolized virus to infect cells and lymphoid tissues of the upper respiratory tract, MV-infected cells were not detected in either the tonsils or the adenoids until after onset of viremia. These data strongly suggest that in our model MV entered the host at the alveolar level by infecting macrophages or dendritic cells, which traffic the virus to BALT or regional lymph nodes, resulting in local amplification and subsequent systemic dissemination by viremia.

## Author Summary

Measles remains an important vaccine-preventable cause of morbidity and mortality in developing countries. The causative agent, measles virus (MV), is one of the most contagious viruses known. Measles has an incubation time of approximately two weeks, and surprisingly little is known about the early events after MV infection. Epithelial cells in the upper respiratory tract have long been considered as early target cells, but more recently alveolar macrophages (AM) and dendritic cells (DC) have been proposed as alternatives. We have infected cynomolgus macaques with a high dose of a recombinant EGFP-expressing MV strain via aerosol inhalation, to ensure that the virus had access to the entire respiratory tract. At 2 days post-infection, MV-infected mononuclear cells were detectable in the alveolar lumen but not in the upper respiratory tract. These infected cells migrated through the bronchus-associated lymphoid tissue to the draining tracheo-bronchial lymph node at 3 days post-infection. Systemic infection was initiated from this point, as observed in macaques euthanized 4 or 5 days post-infection. Thus, even though the aerosolized virus had access to epithelial cells and lymphoid tissues along the entire respiratory tract, AM and DC in the lungs were the first cells that sustained MV replication.

## Introduction

Measles virus (MV) is one of the most contagious human viruses known, and is transmitted via aerosols or by direct contact with contaminated respiratory secretions. Clinical signs appear approximately two weeks after infection and include fever, rash, cough, coryza and conjunctivitis [1]. Measles is associated with a transient but profound immunosuppression, resulting in increased susceptibility to opportunistic infections. While significant progress has recently been made in global control programs, 164,000 deaths were still attributed to measles in 2008 [2].

It has been well established that MV infects cells via receptor-dependent fusion of the virion at the plasma membrane [3]. Two cellular receptors for MV have been identified. In 1993 the membrane cofactor protein CD46, expressed by virtually all nucleated human cells, was the first protein to be identified as MV receptor [4], [5]. However, it soon became evident that only vaccine and laboratory-adapted MV strains were able to utilize this molecule as an entry receptor [6]. Signaling lymphocyte activation molecule (SLAM or CD150), a membrane glycoprotein expressed on subsets of immune cells, was identified as the receptor for wild-type MV strains in 2000 [7], [8]. It is now generally accepted that pathogenic wild-type MV strains use CD150 as high affinity cellular receptor, whereas vaccine and laboratory-adapted strains can use either CD46 or CD150. Distribution of CD150 explains most, but not all aspects of measles pathogenesis and it may be possible that the utilization of additional low-affinity cellular receptors explains how wild-type viruses enter CD150^−^ epithelial or neuronal cells [9]–[11].

Previously, we successfully infected macaques with a recombinant MV based on the pathogenic IC323 strain [12] that expresses enhanced green fluorescent protein (EGFP) from a promoter-proximal additional transcription unit (ATU); this wild-type recombinant virus (rMV^IC323^EGFP) uses CD150 but not CD46 as a cellular entry receptor *in vitro* [13]. In macaques, rMV^IC323^EGFP proved to be virulent and CD150-expressing lymphocytes and dendritic cells (DC) were identified as the predominant target cells for MV replication [14]. In a more recent study, macaques were infected with a rMV^IC323^ that inefficiently binds CD150, showing that this virus was attenuated and indicating that CD150-mediated entry is indeed essential for MV to be fully virulent *in vivo* [15].

*In vitro* studies have demonstrated that at a high multiplicity of infection (MOI) wild-type MV can infect cells that do not express CD150, although this process is inefficient and usually does not result in cell-to-cell spread or virus release [13]. However, a number of CD150^−^ cell types of epithelial or neuronal origin have been identified in which wild-type MV infection at a low MOI results in cytopathic effects and virus release [9]–[11]. It is thought that entry into these CD150^−^ cells is mediated by an unidentified cellular receptor for MV, which is often referred to as the epithelial cell receptor (epR). Even though the receptor has not been identified, epR-binding sites on the MV hemagglutinin protein have been mapped [9], [10], and the receptor appears to be a protein expressed on the basolateral side of polarized epithelial cells associated with tight junctions [9], [16], [17]. In human tissues, cells within the epithelium have historically been shown to be infected by wild-type MV. More recently, epithelial cell infection has been demonstrated with dual immunofluorescence, using the recombinant rMV^IC323^EGFP strain in non-human primates [14], [16]. However, the limited epithelial cell infection observed predominantly occurs in the presence of substantial infection of lymphoid and myeloid cells, which is consistent with the differential expression of CD150 on these cell types.

It has been postulated that MV infection starts from the luminal side of the upper respiratory epithelium [1]. However, there is no direct evidence for initial MV infection and replication in epithelial cells. Furthermore, the absence of CD150 or epR on the apical side of these cells makes it highly unlikely that respiratory epithelial cells are an initial target for MV. However, the respiratory epithelium contains many other cell types besides epithelial cells. Several research groups have postulated new strategies for MV to enter a host, namely via direct initial infection of CD150^+^ immune cells present throughout the respiratory tract and interdigitated within the epithelium [14], [18]. In 2006, the C-type lectin DC-SIGN was identified as an attachment receptor for MV [19]. *In vitro* DC-SIGN expressing DC could efficiently capture and transmit MV to CD4^+^ and CD8^+^ T-lymphocytes expressing CD150. This suggests a potential role for the DC as an initial target cell *in vivo*, where DC capture the virus from the luminal side of the respiratory tract and, with or without productive infection, transport the virus to draining lymph nodes (LN) containing many CD150^+^ cells, thereby initiating the typical systemic infection [19], [20].

In the present study, we have generated a rMV based on a genotype B3 wild-type MV isolate from Khartoum, Sudan. The open reading frame (ORF) encoding EGFP was introduced into the virus genome in the promoter-proximal position within an ATU using a similar approach as previously described for rMV^IC323^EGFP [12]. In an attempt to identify the early target cells of MV in non-human primates, macaques were infected with rMV^KS^EGFP and sacrificed 2, 3, 4 or 5 days post-infection (d.p.i). Infections were performed by inhalation of a high dose of virus formulated as small-particle size nebulized aerosol, thus exposing the entire upper and lower respiratory tract to the virus. MV-infected EGFP^+^ cells were identified at all four time points, albeit at low levels 2 and 3 d.p.i. Infection is initiated in large mononuclear cells in the alveolar lumen, most likely either AM or DC.

## Results

### Generation and characterization of rMV^KS^EGFP

**Figure 1:**
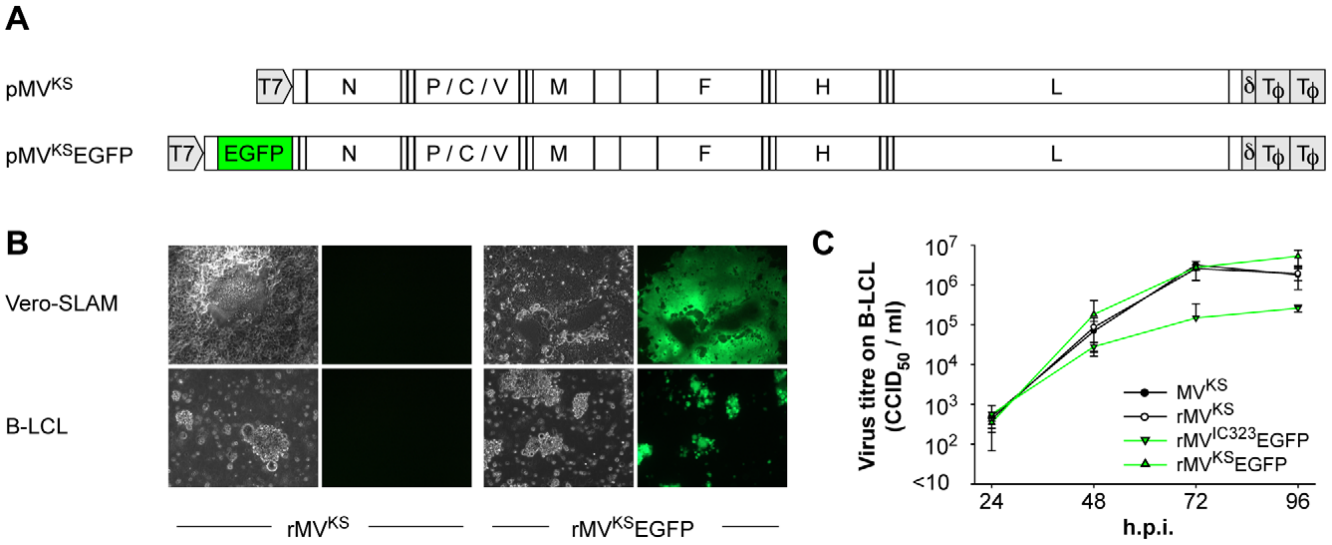
Generation and growth of rMV^KS^EGFP. (A) Plasmids generated after RT-PCR, cloning and sequencing of MV RNA isolated from MV^KS^-infected PBMC. pMV^KS^ is a full-length plasmid containing the complete antigenome of MV^KS^ and pMV^KS^EGFP was modified by the insertion of an ATU at the promoter proximal position containing the ORF encoding EGFP. (B) rMV^KS^ and rMV^KS^EGFP were rescued from Vero-SLAM cells and passaged in B-LCL. Fluorescence microscopy confirmed high levels of EGFP expression in rMV^KS^EGFP infected cells. (C) Growth curves of MV^KS^, rMV^KS^, rMV^KS^EGFP and rMV^IC323^EGFP in human B-LCL. Virus was harvested 24, 48, 72 and 96 hours post infection, CCID_50_ was determined in an endpoint titration test. Measurements shown are averages of triplicates ± SD. Key: h.p.i.: hours post infection.

MVi/Khartoum.SUD/34.97/2 (MV^KS^) was isolated from a measles case in Khartoum, Sudan in 1997 [21]–[23]. This virus was previously shown to be highly virulent in macaques [24]. A consensus sequence of the complete viral genome was derived *de novo*, including the 3′ and 5′ ends which were sequenced following rapid amplification of cDNA ends (RACE), and a full-length anti-genomic plasmid (pMV^KS^) was constructed (figure 1A). The plasmid was modified by the addition of an ATU encoding EGFP at the promoter proximal position (figure 1A) to generate pMV^KS^EGFP. Recombinant viruses rMV^KS^ and rMV^KS^EGFP were recovered following transfection of Vero-SLAM cells, and were passaged exclusively on Epstein-Barr virus-transformed human B-lymphoblastic cells (B-LCL) (figure 1B). Presence of a silent point mutation in the MV nucleocapsid (N) ORF (T1245C) acts as a genetic tag and its presence was confirmed by RT-PCR and sequencing of the ORF (data not shown). Observation of rMV^KS^EGFP by fluorescence microscopy revealed a high level of EGFP expression associated with single infected cells and multinucleated syncytia. Growth analysis of MV^KS^, rMV^KS^, rMV^KS^EGFP and rMV^IC323^EGFP in B-LCL over a period of 4 days demonstrated that the viruses reached equivalent titers (figure 1C).

### Early rMV^KS^EGFP replication in the respiratory tract

**Figure 2:**
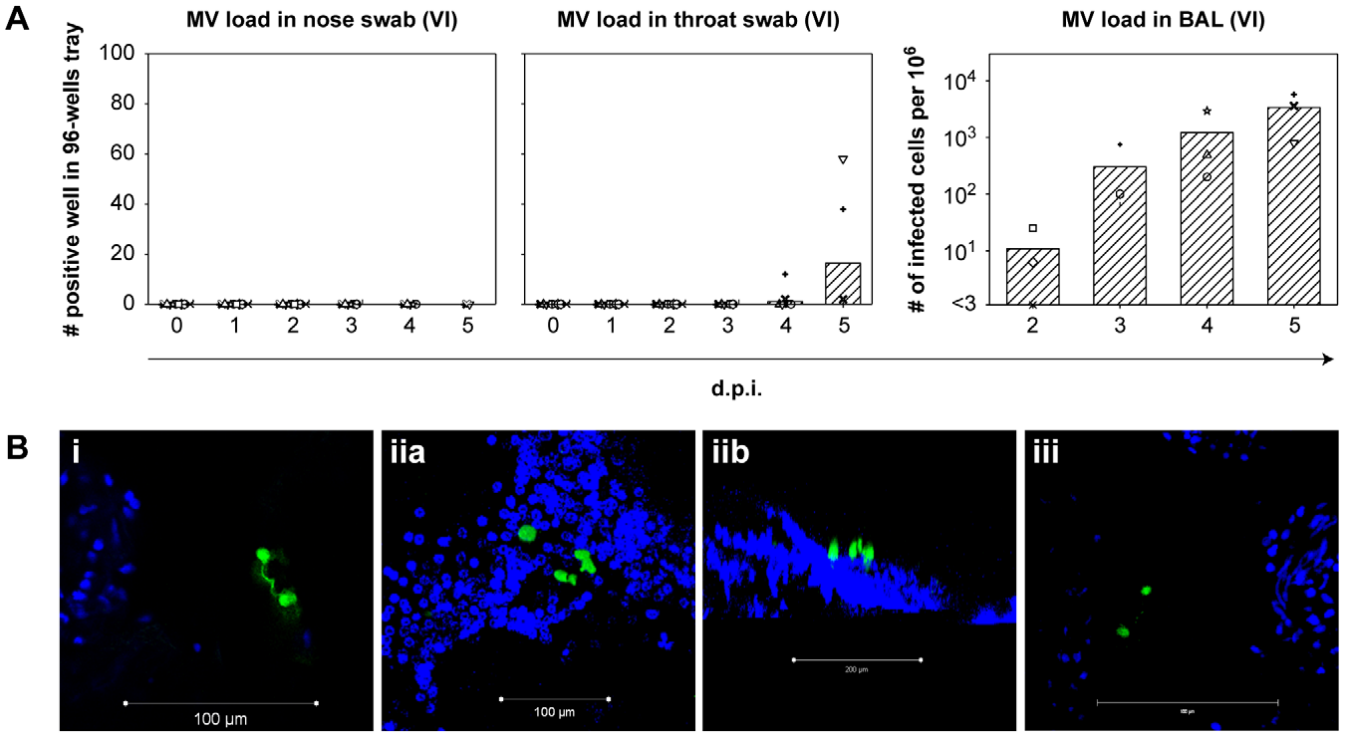
Early rMV^KS^EGFP replication in the respiratory tract. (A) Virus isolation performed from nose and throat swabs (left two panels), and from BAL cells (right panel). Each symbol represents an individual animal, bars indicate the geometric mean. Key: VI: virus isolation; d.p.i.: days post infection. (B) Live cell confocal microscopy performed on agarose-inflated lung slices from animals on 2 and 3 d.p.i. EGFP^+^ cells are shown in green, DAPI was used to counter stain nuclei (blue). Three images were collected, labeled i, ii and iii. Panels iia and iib show infected cells in one image from different orientations. Matching 3D-videos for (Bi, Bii and Biii) are available as supporting data.

Four groups of three cynomolgus macaques were infected with rMV^KS^EGFP via the aerosol route as described previously [25]. Throat and nose swabs were collected daily and virus isolations were performed to determine the MV load in these clinical samples. Necropsies were performed 2, 3, 4 and 5 d.p.i. BAL cells were collected for virus isolation and the entire respiratory tract was screened macroscopically and microscopically for fluorescence by live cell UV fluorescence and confocal scanning laser microscopy. At 2 and 3 d.p.i., no macroscopic fluorescence was detectable, probably because of low levels of viral replication. No virus could be isolated from the nose, whereas from throat swabs virus was only isolated at 4 (2/6 animals) and 5 (3/3 animals) d.p.i. (figure 2A). However, MV was isolated from BAL cells as early as 2 d.p.i. (2/3 animals) and by 3, 4 and 5 d.p.i. virus was isolated from BAL cells of all animals with virus loads increasing over time (figure 2A). Microscopic detection of MV replication in freshly collected tissues of the respiratory tract proved that as early as 2 d.p.i. the virus was consistently present in the lungs of all animals (figure 2B and supporting videos S1, S2 and S3). On 2 and 3 d.p.i. single infected mononuclear cells with the appearance, size and typical tissue distribution of AM and/or DC were detected attached to the alveolar wall or inside the alveolar lumen. At these time points no MV-infected epithelial cells were detected in the lungs of any animal, either phenotypically, following screening of lung slices for EGFP-positive cells or histologically, by dual staining of MV proteins and cytokeratin. MV-infected cells could not be detected in the nasal septum, nasal concha, nasal lining, trachea or primary bronchus 2 and 3 d.p.i. By 4 d.p.i. a fluorescent signal was detected in the nasal septum of a single animal, and by 5 d.p.i. the nose, trachea and primary bronchus were consistently positive (table 1).

**Table 1:** Dissemination of MV in tissues during early stage of infection.

	Days post-infection (d.p.i.)			
Tissue	2	3	4	5
Lung	**3**	**3**	**3**	**3**
Tracheobronchial LN^1^	**1**	**3**	**3**	**3**
PBMC^2^	**-** 4	**-**	**3**	**3**
Adenoid	**1**	**-**	**3**	**3**
Retropharyngeal LN	**-**	**-**	**1**	**3**
Mandibular LN	**-**	**-**	**-**	**3**
GALT^3^	**-**	**-**	**1**	**3**
Spleen	**-**	**-**	**1**	**2**
Tonsil	**-**	**-**	**1**	**1**
Thymus	**-**	**-**	**1**	**1**
Nasal septum	**-**	**-**	**1**	**1**
Nasal concha	**-**	**-**	**-**	**2**
Trachea	**-**	**-**	**-**	**2**
Inguinal LN	**-**	**-**	**-**	**2**
Axillary LN	**-**	**-**	**-**	**1**
Mesenteric LN	**-**	**-**	**-**	**1**

Numbers indicate the number of macaques with EGFP^+^ cells in this tissue (n = 3).

1 LN: lymph node.

2 PBMC: peripheral blood mononuclear cells.

3 GALT: gut-associated lymphoid tissue.

4 -: no EGFP^+^ cells detected.

### Systemic rMV^KS^EGFP replication

**Figure 3:**
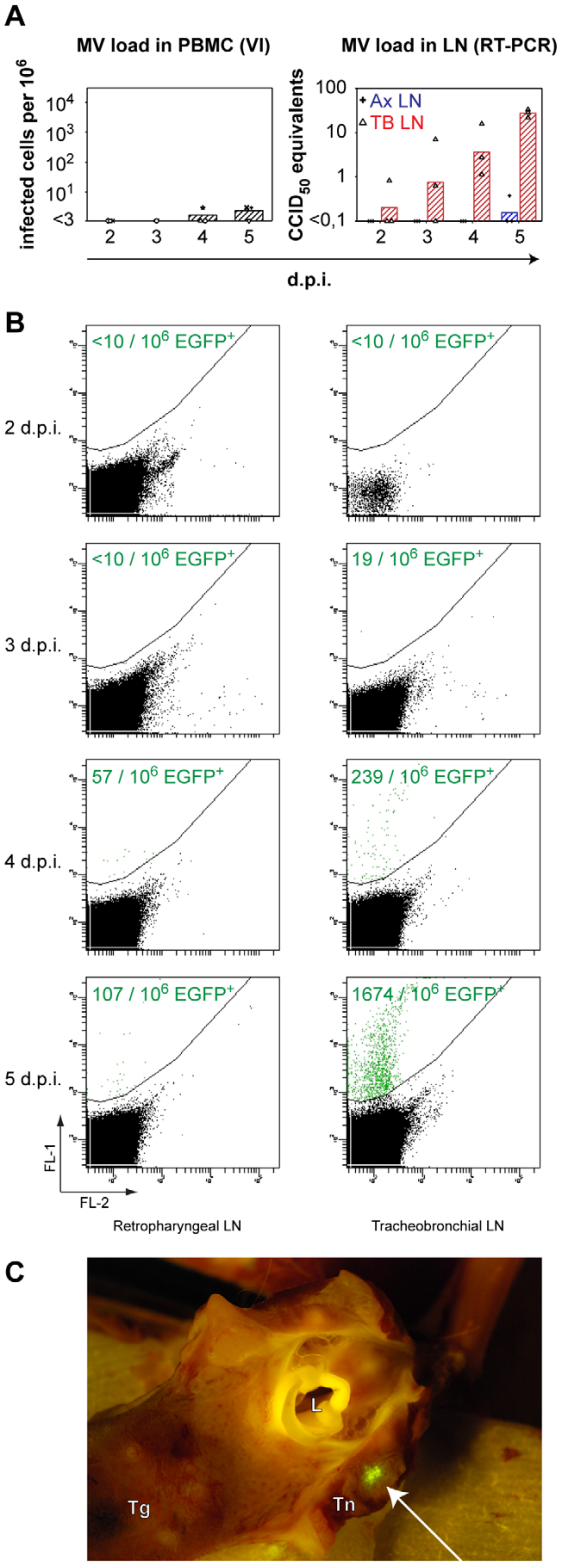
Systemic rMV^KS^EGFP replication. (A) MV load in PBMC and LN. The left panel shows virus isolations performed from PBMC, each symbol represents an individual animal, bars indicate the geometric means. The right panel shows the presence of MV genome in the axillary LN (crosshairs, geometric mean in blue) and in the tracheobronchial LN (triangles, geometric mean in red). Key: VI: virus isolation; RT-PCR: real-time reverse transcriptase PCR; d.p.i.: days post infection. (B) Detection of EGFP^+^ cells by flow cytometry from the retropharyngeal LN (left) and the tracheobronchial LN (right) on 2, 3, 4 and 5 d.p.i. Data are shown as dot plots of FL-1 (EGFP) versus FL-2 (empty channel), generated with BD FACSDiva software. In these plots autofluorescent cells usually appear on a diagonal line as they cause comparable signals in both channels. The EGFP-positive events were gated as indicated by the curvilinear line. Data of a representative animal are shown on each time point. Numbers of EGFP^+^ cells per million total cells are shown in each plot. (C) Representative example of macroscopic EGFP detection at 5 d.p.i. Arrow indicates the infected tonsillar tissue expressing EGFP. Key: Tg: tongue; Tn: tonsil; L: larynx.

Virus isolations were performed from peripheral blood mononuclear cells (PBMC) and single cell suspensions of four lymphoid organs (retropharyngeal LN, mandibular LN, tonsil and tracheo-bronchial LN). RNA isolations and virus detection by RT-PCR were performed on the axillary LN and tracheo-bronchial LN, which drain the arm and the lungs, respectively. Furthermore, PBMC and all lymphoid organs were analyzed directly by flow cytometry and UV microscopy for fluorescence. Viremia was detected in all animals on 4 and 5 d.p.i. but in none of the animals sampled 2 and 3 d.p.i. (figure 3A, left panel, and table 1). Virus was not isolated from any lymphoid organ 2 d.p.i. However, by 3 d.p.i. virus was isolated from the tracheo-bronchial LN of all animals (data not shown). RT-PCR and flow cytometry confirmed the early presence of MV in the tracheo-bronchial LN, but not in more distally located LN, for example the axillary and retropharyngeal LN (figure 3A, right panel and 3B). Flow cytometry confirmed that the number of EGFP^+^ cells increased over time. Virus was detected by almost all methods in multiple lymphoid organs 4 and 5 d.p.i. by which time the MV was spreading systemically (table 1). Macroscopic detection of EGFP proved possible only 4 and 5 d.p.i (figure 3C). The tonsil of a single animal was positive 4 d.p.i . By 5 d.p.i. MV was detected macroscopically at multiple locations (adenoids, tonsil, retropharyngeal LN, trachea, tongue, tracheo-bronchial LN) in all animals indicating widespread dissemination. Phenotyping of the MV-infected cells in PBMC or single cell suspensions of lymphoid tissues collected on 4 and 5 d.p.i. showed that these were predominantly T- or B-lymphocytes (data not shown).

### Phenotyping of early MV-infected cells in the lungs

**Figure 4:**
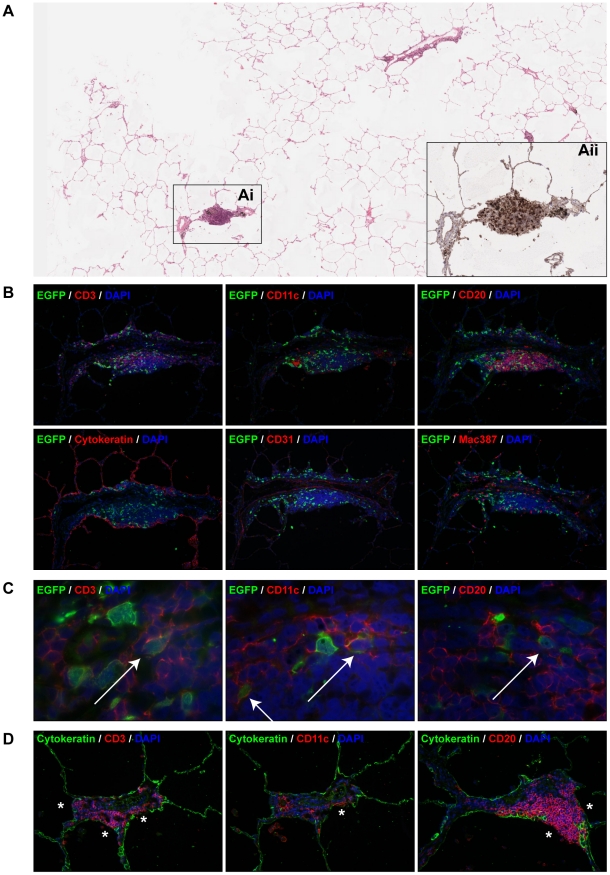
Characterization of MV infection in BALT structures. (A) H&E staining on lung slice from an animal euthanized on 3 d.p.i.. The number of EGFP^+^ foci was extremely low, the boxed area (Ai) is a BALT which was the only area on the section where EGFP^+^ cells were present. (Aii) shows a serial section stained with anti-GFP (black) to detect the presence of virus (see also Figure S3, annotated immunohistochemical and H&E annotated pathology scans). (B) Indirect dual immunofluorescence of the infected BALT structure, showing the presence of T-lymphocytes (CD3), DC or macrophages (CD11c, mac387) and B-lymphocytes (CD20) within the BALT. The BALT is lined by a layer of cytokeratin-positive epithelial cells, and has a blood vessel with CD31-positive endothelium running through it transversely. (C) Higher magnifications of dual immunofluorescence within the BALT indicates the presence of MV-infected T-lymphocytes (CD3), DC or macrophages (CD11c) and B-lymphocytes (CD20), Double positive cells are indicated by arrows. In panel (B) and (C), EGFP^+^ cells are shown in green, cell-type specific staining is shown in red. DAPI was used to counter stain nuclei in blue. (D) Dual immunofluorescence performed on uninfected BALT region. Dual labelling with cytokeratin (green) and CD3, CD11c or CD20 (red) showed that T-lymphocytes, B-lymphocytes and DC or macrophages are present in very close proximity or in direct contact with the alveolar or bronchiolar lumen (asterisks). Single colour images for (C) are available as supporting data (figure S2).

Early after infection MV was consistently present in the lungs. In order to characterize the early target cells, live agarose-inflated lung slices containing EGFP^+^ cells were formalin-fixed and paraffin-embedded. Serial sections were cut and used for immunohistochemistry and indirect immunofluorescence to determine the precise location of MV infection, identify the phenotype of the infected cells and gain an understanding of how such “seeding” of MV infection in the lungs might lead to the establishment of systemic infection. At 3 d.p.i., two foci of infection were identified in paraffin-embedded lung sections of one of the three infected animals, interestingly both in BALT (figure 4A and supporting online, annotated immunohistochemical and H&E pathological scans figure S3). These BALT structures were lined by a cytokeratin-positive epithelial cell layer and contained numerous immune cells (figure 4B), that stained positive with CD11c for AM or DC, Mac387 for macrophages, CD20 for B-lymphocytes and/or CD3 for T-lymphocytes. Blood vessels, identified using the endothelial cell-specific marker CD31, were always present in BALT structures irrespective of the presence or absence of MV-infected cells. Since there were a limited number of foci of infection identified this early after infection, we were unable to quantify the levels of infection in different cell types. However, MV-infected B-lymphocytes, T-lymphocytes and DC could readily be detected by specific dual labeling at higher magnifications (figure 4C and Figure S2). Multiple foci of MV-infected cells were detected 4 and 5 d.p.i. in the alveolar lumina and walls of all animals and the majority of these infected cells were CD11c^+^ (Figure S1), consistent with what had been observed previously in macaques euthanized 7 d.p.i. [25].

Analysis of BALT structures in the lungs of non-infected macaques indicated that even though cytokeratin-positive epithelial cells lined these structures, cells of lymphoid and myeloid origin were present both within the epithelium and in direct contact with the adjacent lumen. Indirect immunofluorescence identified CD11c^+^, CD3^+^ and CD20^+^ cells in direct contact with the lumen of alveoli, bronchioles or bronchi (figure 4D, asterisks). This was also confirmed in virus-negative BALT structures from uninfected animals (supporting online, annotated immunohistochemical and H&E pathological scans S3).

### Early MV infection in lymphoid tissue is frequently associated with the presence of blood vessels

**Figure 5:**
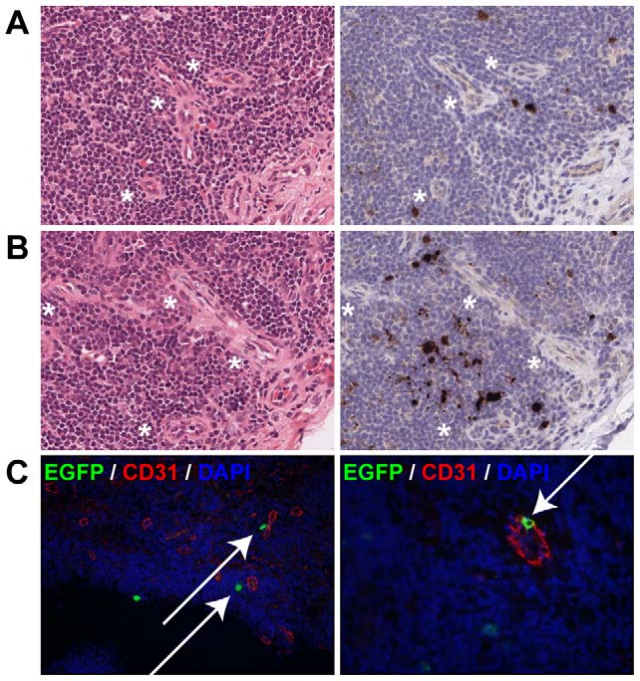
Dissemination of MV into the lymphoid organs via blood vessels. (A, B) H&E staining (left panel) and EGFP staining (right panel) on serial sections of tonsils at 4 d.p.i. (A) and 5 d.p.i. (B). Asterisk denote the proximity of venules to MV-infected cells. (C) Dual labeling of EGFP (green) and the endothelial marker CD31 (red) performed on the tonsils from animals euthanized 5 d.p.i. The left panel shows MV-infected cells in close proximity to CD31^+^ endothelial cells of venules (arrows), the right panel shows an MV-infected cell migrating through the wall of the venule (arrow). DAPI was used to counter stain nuclei in blue. Single color images for (C) are available as supporting data (figure S2).

Within the infected BALT structures, MV-infected cells were readily detected in direct contact with or in close proximity to the endothelial wall of blood vessels. A similar distribution was observed in the tracheo-bronchial LN, tonsils and adenoids on 4 and 5 d.p.i., in which MV-infected cells were mostly detected in close proximity to venules (figure 5). On rare occasions, MV-infected cells with the morphology of dendritic cells could be seen migrating through the endothelium (figure 5, right panel and figure S2).

## Discussion

In the present study, we have generated and utilized a virulent rMV strain expressing EGFP, based on a wild-type genotype B3 MV isolate from Khartoum, Sudan. In growth curves in human B-LCL recombinant strains rMV^KS^ and rMV^KS^EGFP reached equivalent titers, which were slightly higher than those reached by rMV^IC323^EGFP. These data suggest that the addition of EGFP into the genome had no detectable effect on virus fitness as determined *in vitro*. Pathogenesis studies performed with molecular clones of wild-type MV have thus far exclusively been based on the Japanese strain IC323 [12]. Development of a second recombinant wild-type MV serves to complement ongoing studies of MV pathogenesis and ensures that observations are not strain-specific. Expression of EGFP from a promoter-proximal ATU leads to significant amounts of EGFP and, interestingly in the case of MV, has no or only a limited effect on the virulence *in vivo*. This was not the case for other morbilliviruses, for example canine distemper virus [26]. The new recombinant virus rMV^KS^EGFP described here also proved to be virulent in cotton rats [Lemon K, manuscript in preparation], and allowed sensitive microscopic detection of the virus *in vitro*, *ex vivo* and *in vivo*.

Macaques were infected with a high dose of rMV^KS^EGFP via the aerosol route and early necropsies were performed to identify the initial target organs, tissues and cells. The nebulizer used was similar to the type that is used in ongoing clinical trials of measles aerosol vaccination, organized in India by the World Health Organization. The nebulizer produced a volume median diameter (VMD) of 4–6 µm, allowing the inoculum to deposit both in the upper respiratory and lower respiratory tract, and reach the alveolar lumina. A high infectious dose (10^6^ CCID_50_) was nebulized to ensure that all potential early target cells for MV infection in the respiratory tract were exposed to the virus. Such an approach is important in any study which aims to identify key cells targeted by a respiratory virus as it ensures the pathogen can access the broadest range of cell types and associated tissues throughout the respiratory tract. However, it is important to acknowledge our limited understanding of how MV is transmitted from human to human: in our current study we have infected animals with cell-free virus but transmission between humans could also involve excretion of cell-associated virus. Studies which examine the pathological consequences of MV infection in animals at later time points would greatly facilitate our understanding of virus transmission, both for MV and other respiratory viruses. The techniques and bank of tissues collected in this and other studies could be used to shed light on person to person transmission.

Our data strongly suggest the following sequence of events. At early time points (2 and 3 d.p.i.), MV infected large mononuclear cells with the phenotype and location of AM or DC. Targeting of these cells was followed by the establishment of localized MV replication in close proximity, lymphoid aggregates in the lungs (BALT). These BALT structures contained a large number of B-cells and memory CD4^+^ T-cells [27], both cell types previously described as preferential targets for MV in lymphoid tissue at later time points [14]. Seeding and amplification of the infection in these microenvironments, which are well suited to a lymphotropic virus such as MV, is likely to be critical in the establishment of the infection. From the lungs, MV was transported by infected cells to the draining tracheo-bronchial LN. After localized replication in the lungs and increased replication in the tracheo-bronchial LN, MV spread systemically through viremia to the majority of lymphoid organs by 4 or 5 d.p.i. MV-infected cells were always detected in close proximity to venules within lymphoid organs, suggesting that these were involved in spreading the virus.

It has been stated that MV initially targets the epithelium of the upper respiratory tract to establish infection [1]. However, all known wild-type MV receptors are absent on the luminal side of respiratory epithelial cells, making their initial infection by MV highly unlikely. Other potential entry strategies by which MV might enter a susceptible host have been described in the literature. For example, the Trojan horse strategy that has been described for HIV-1 has also been considered for MV [20]. DC could capture MV from the respiratory tract using dendrites protruding through the epithelium and transmit virus to CD4^+^ and CD8^+^ T-lymphocytes, leading to infection. *In vivo* in the macaque model, infection of DC has indeed been described in submucosal tissues [14]. Furthermore, in the present study we demonstrate that MV was detectable in the lungs 2 d.p.i., since MV-infected cells could be both isolated from BAL and imaged *in situ* by live cell confocal scanning laser microscopy. These data confirm that large mononuclear cells present in the alveolar lumen or lining the alveolar epithelium, most likely AM and/or DC, are among the earliest cells infected by MV in the macaque model. Even though the Ifnar^KO^-SLAMGe mouse model does not recapitulate the whole spectrum of measles pathogenesis, initial infection of AM and DC was also shown in this model by flow cytometry [28].

We show here that, in the respiratory tract, BALT structures were the only MV-infected tissues at 3 d.p.i. BALT is normally lined by a continuous epithelial layer, making direct entry of MV unlikely. However, the epithelium of the BALT has previously been described to be a flattened respiratory epithelium, with common influx and efflux of lymphocytes, AM and DC [29]. Furthermore, the epithelium of BALT of many mammalian species contains M-cells [30], cells that are specialized for antigen uptake. In mouse models, it has been shown that BALT plays a role in the uptake of multiple bacteria (*Pseudomonas aeruginosa* [31], *Mycobacterium tuberculosis* [32]). Reoviruses have also been described to be taken up by M-cells, with subsequent spread to the regional lymph nodes [33]. In this study we did not observe antigen uptake by M-cells. Instead, we observed infected cells resembling AM or DC at 2 d.p.i. and suggest that they transported MV through the BALT epithelium into the underlying lymphoid tissue.

An alternative route for MV to enter a susceptible host would be via direct infection of CD150^+^ cells in Waldeyer's tonsillar ring, consisting of tonsils and adenoids. Tonsils and adenoids are lined by CD150^−^ epithelial cells, but at sites of damage or in tonsillar crypts direct infection of CD150^+^ cells at the luminal surface might be possible. In our model, tonsils and adenoids were directly exposed to a high dose of nebulized virus, but a consistent level of infection was only detected 4 and 5 d.p.i., when the infection already was systemic. Only one out of six animals had MV-infected cells in the adenoid 2 d.p.i. and no infection was observed in the tonsils 2 and 3 d.p.i. These data suggest that MV cannot easily penetrate the epithelial layer to initiate MV infection of CD150^+^ cells in tonsillar tissue of the Waldeyer's ring.

Following the initial infection of cells in the lung, the draining TB-LN was the first lymphoid organ being consistently MV-positive 3 d.p.i. Since this LN drains the lungs, it is most likely that MV-infected cells are transported through lymphatic vessels to reach the TB-LN. In the BALT and TB-LN, MV-infected cells were often detected in close proximity of venules. We hypothesize that MV-infected cells are transported through these venules into the bloodstream, from where they reach the spleen and other lymphoid organs, initiating the systemic infection as observed 4 and 5 d.p.i. The proximity of MV-infected cells to venules in the tonsils and adenoids 4 and 5 d.p.i. substantiates this hypothesis.

In conclusion, aerosol exposure of the entire respiratory tract of macaques to a high dose of infectious MV leads to initial infection of mononuclear cells in the alveoli (2 d.p.i.), followed by MV replication in BALT (3 d.p.i.). Phenotypically and based on location it is likely that the initial target cells in the alveoli are AM or DC. In BALT, T-lymphocytes, B-lymphocytes and DCs are all productively infected. CD11c^+^ cells are the major target cell population in the lungs 4 and 5 d.p.i. indicating an important role for AM and/or DC early in establishing the infection.

## Materials and Methods

### Ethics statement

Animals were housed and experiments were conducted in strict compliance with European guidelines (EU directive on animal testing 86/609/EEC) and Dutch legislation (Experiments on Animals Act, 1997). The protocol was approved by the independent animal experimentation ethical review committee DCC in Driebergen, the Netherlands (Erasmus MC permit number EUR1664). Animal welfare was observed on a daily basis, and all animal handling was performed under light anesthesia using a mixture of ketamine and medetomidine to minimize animal suffering. After handling atipamezole was administered to antagonize the effect of medetomidine.

### Generation of wild-type recombinant MV expressing EGFP

rMV^KS^EGFP is based on a wild-type genotype B3 virus isolated from PBMC collected in 1997 from a severe measles case in Khartoum, Sudan [21]. The clinical isolate (MV^KS^) was passaged exclusively in CD150^+^ human B-LCL and was previously shown to be highly pathogenic in macaques [24]. Total RNA was isolated from B-LCL infected with MV^KS^ and the complete consensus genomic sequence determined following RT-PCR (GenBank accession number HM439386). The sequences of the genomic termini were confirmed by 5′ RACE. A full-length cDNA which expressed the MV^KS^ anti-genome (pMV^KS^) was constructed based on a modified pBluescript vector [34]. A single silent mutation was introduced into the N ORF (T1245C) to act as a genetic tag to distinguish recombinant virus from the clinical isolate. The full-length plasmid was modified further by the introduction of an ATU expressing EGFP at the promoter proximal position to generate pMV^KS^EGFP. Plasmid sequences are available on request. Recombinant viruses were recovered from MVA-T7-infected Vero-SLAM cells transfected with the full-length plasmids along with plasmids expressing MV N, P and L. Virus stocks were grown in B-LCL and tested negative for contamination with *Mycoplasma* species. Virus titers were determined by endpoint titration in Vero-SLAM cells, and expressed in 50% cell culture infectious dose (CCID_50_).

Virus fitness of MV^KS^, rMV^KS^, rMV^KS^EGFP and rMV^IC323^EGFP was compared in a growth curve. Human B-LCL were infected in triplicate with MV^KS^, rMV^KS^, rMV^KS^EGFP or rMV^IC323^EGFP in 24-wells plates at MOI 0.1. At 24, 48, 72 and 96 hours post infection plates were freeze-thawed at −80°C, and cells and supernatant fluids were harvested. After sonification and clarification, the amounts of cell-free virus at different time-points were determined by endpoint titration in Vero-CD150 cells using ten-fold dilutions and testing eight wells per dilution, and expressed in CCID_50_.

### Early target cell animal study

Twelve juvenile, MV-seronegative cynomolgus macaques (*Macaca fascicularis*) were housed in negatively pressurized, HEPA-filtered BSL-3 isolator cages. Animals were infected with rMV^KS^EGFP by aerosol inhalation using a pediatric face mask (ComfortSeal silicone mask assembly, small, Monaghan Medical Corp., Plattsburgh NY). Aerosol was generated using the Aerogen Aeroneb Lab nebulizer with an OnQ aerosol generator (kind gift of Dr. J. Fink, Aerogen) as previously described [25]. This nebulizer generates a small particle size aerosol (VMD 4–6 µm), which is deposited on epithelia throughout the entire respiratory tract upon inhalation [35]. A total dose of 10^6^ CCID_50_ was nebulized, but we previously found that a substantial part of nebulized virus is lost due to inactivation during nebulization, condensation in the nebulizer tubing or face mask, deposition on the skin of the animals or deposition in the mouth followed by swallowing. We therefore estimated that the delivered dose was approximately 10^5^ CCID_50_, of which based on previous studies approximately 10% is expected to reach epithelia in the lungs [35]. Animals were euthanized on 2, 3, 4 or 5 d.p.i. (n = 3 per time point).

### Necropsy

Animals were euthanized by sedation with ketamine (20 mg/kg body weight) followed by exsanguination. Macroscopic detection of EGFP was performed at necropsy as described previously [14]. Briefly, fluorescence was detected with a custom-made lamp containing 6 LEDs (peak emission 490–495nm); emitted fluorescence was detected through an amber cover of a UV transilluminator used for screening DNA gels. Photographs were made using a Nikon D80 SLR camera. Organs were collected in PBS, directly processed and screened for presence of EGFP by UV microscopy. From here, EGFP^+^ samples were transferred to 4% (w/v) paraformaldehyde in PBS (to preserve EGFP autofluorescence) or to 10% neutral buffered formalin. The left lung lobe was inflated as described previously [Lemon K, manuscript in preparation] using a solution of 4% (w/v) agarose in PBS mixed 1∶1 with DMEM/Ham's F12 medium supplemented with L-glutamine (2 mM), 10% (v/v) heat-inactivated fetal bovine serum (FBS), penicillin (100 U/ml) and streptomycin (100 µg/ml). The inflated lung was allowed to solidify on ice, and ∼1 mm slices were cut by hand. Slices were permeabilized with 0,1% (v/v/) Triton-X100, counterstained with DAPI and directly analyzed for EGFP fluorescence by confocal laser scanning microscopy with a LSM700 system fitted on an Axio Observer Z1 inverted microscope (Zeiss). Images and videos were generated using Zen software.

### Blood samples

Small volume blood samples were collected in Vacuette tubes containing K_3_EDTA as an anticoagulant daily after infection. White blood cells (WBC) were obtained by treatment of EDTA blood with red blood cell lysis buffer (Roche diagnostics, Penzberg, Germany) and used directly for detection of EGFP by flow cytometry. During necropsy blood was collected in heparin to prevent coagulation, PBMC were isolated by density gradient centrifugation, washed, resuspended in complete RPMI-1640 medium (Gibco Invitrogen, Carlsbad, CA, USA) supplemented with L-glutamine (2 mM), 10% (v/v) heat-inactivated FBS, penicillin (100 U/ml) and streptomycin (100 µg/ml), counted using a haemocytometer and used directly for flow cytometry and virus isolation. Isolation of MV was performed on human B-LCL using an infectious center test as previously described [24]. Virus isolations were monitored by UV microscopy for EGFP fluorescence after co-cultivation with B-LCL for 3–6 days and results were expressed as number of virus-infected cells per 10^6^ total cells.

### Broncho-alveolar lavage

A BAL was performed post-mortem by direct infusion of 10 ml PBS into the right lung lobe. BAL cells were resuspended in culture medium with supplements as described above, counted and used directly virus isolation. Virus isolation was performed on B-LCL as described above. The remaining BAL cells were examined for EGFP expression by UV microscopy.

### Throat and nose swabs

Throat and nose swabs were collected daily in transport medium (EMEM with Hanks' salts, supplemented with lactalbumine enzymatic hydrolysate, penicillin, streptomycin, polymyxine B sulphate, nystatin, gentamicin and glycerol) and frozen at −80°C. After thawing samples were vortexed, the swab was removed and the remaining transport medium was used for virus isolation [36]. Isolation of MV was performed on Vero-SLAM cells using an infectious center test as previously described [24]. The isolations were screened for EGFP fluorescence at day 3 and 7 post titration and results are expressed as the number of EGFP^+^ wells per 96 total wells.

### Lymphoid organs

Lymphoid organs were collected during necropsy in PBS for direct preparation of single cell suspensions using cell strainers with a 100 µm pore size (BD Biosciences). Single cell suspensions were used directly for detection of EGFP by flow cytometry. From a selection of lymphoid organs (retropharyngeal LN, mandibular LN, tonsil and tracheobronchial LN) single cell suspensions were also used for virus isolation on Vero-SLAM cells as described above. The isolations were screened for EGFP fluorescence at day 3 and 7 post titration. The axillary and tracheobronchial LN were also collected in RNA later (Ambion) during necropsy for virus detection by real-time RT-PCR.

### Flow cytometry

Freshly isolated WBC, PBMC and single cell suspensions prepared from lymphoid organs were analyzed unstained for EGFP expression by flow cytometry. EGFP was detected in the FITC channel on a FACS Canto II, approximately 10^6^ events were obtained per sample to allow detection of low frequent EGFP^+^ populations.

### Immunohistochemical and immunofluorescence analysis of formalin-fixed tissues

Only lung slices which were scored positive on live UV fluorescent screening were processed to paraffin. At days 2 and 3, 8/49 and 16/95 slices were scored positive, respectively. Sections (7 µm) were cut and deparaffinized, antigen retrieval was performed in a pressure cooker at full power for 3 min in 0.01 M TRIS-EDTA buffer (pH 9.0). MV-infected cells were detected using a polyclonal rabbit antibody to EGFP (Invitrogen). Sections were incubated in primary antibody overnight at 4°C, and specific antibody-antigen binding sites were detected using an Envision-Peroxidase system with DAB (DAKO) as substrate. Dual labeling indirect immunofluorescence was performed using polyclonal rabbit anti-EGFP and monoclonal mouse antibodies to the macrophage/DC marker CD11c (Novocastra, clone 5D11), the T-lymphocyte marker CD3 (DAKO, clone F7.2.38), the B-lymphocyte marker CD20 (DAKO, clone L26), the epithelial cell marker cytokeratin (DAKO, clone AE1/AE3), the endothelial cell marker CD31 (DAKO, clone JC70A) and the macrophage marker Mac387 (Abcam). Further dual labeling to assess the organization of epithelia and different cell types within BALT were carried out with a polyclonal antibody to epithelial cytokeratin (DAKO, Cat. No. Z0622) in combination with the above monoclonal antibodies to CD3, CD20 or CD11c. In all cases antigen binding sites were detected with a mixture of anti-mouse Alexa 568 and anti-rabbit Alexa 488 (Invitrogen). Sections were counterstained with DAPI hardset mounting medium (Vector). All fluorescently stained slides were assessed and digital fluorescent images acquired with a Leica DFC350 FX digital camera and processed using Leica FW4000 software.

## Supporting Information

Figure S1 CD11c+ DC and macrophages targeted in the lung at 4 and 5 d.p.i. At 4 and 5 d.p.i. the CD11c+ DC or macrophage population was the major cell type in the lung in which MV replicates. Dual labelling for EGFP (green) and CD11c (red), DAPI was used to counter stain nuclei in blue. Left panels show EGFP alone (green), centre panels show CD11c alone (red), right panels show overlay of EGFP and CD11c. The two rows are two representative examples of double positive cells as indicated by arrows.
(2.21 MB TIF)

Figure S2 Single color images for figure 4C and 5C.
(9.75 MB TIF)

Figure S3 On-line immunohistochemical and H&E pathology scans. Four pathology slides were scanned and digitized at a high resolution and annotated.
(0.06 MB PDF)

Video S1 Z-stack as rendered 3D-movie corresponding to figure 2, panel Bi.
(1.91 MB MOV)

Video S2 Z-stack as rendered 3D-movie corresponding to figure 2, panel Bii.
(2.16 MB MOV)

Video S3 Z-stack as rendered 3D-movie corresponding to figure 2, panel Biii.
(1.51 MB MOV)

## References

[1] Griffin DE, Knipe DM, Howley PM (2007). Measles virus.. Fields Virology.

[2] WHO (2009). Global reductions in measles mortality 2000–2008 and the risk of measles resurgence.. Wkly Epidemiol Rec.

[3] Smith EC, Popa A, Chang A, Masante C, Dutch RE (2009). Viral entry mechanisms: the increasing diversity of paramyxovirus entry.. FEBS J.

[4] Dörig RE, Marcil A, Chopra A, Richardson CD (1993). The human CD46 molecule is a receptor for measles virus (Edmonston strain).. Cell.

[5] Naniche D, Varior-Krishnan G, Cervoni F, Wild TF, Rossi B (1993). Human membrane cofactor protein (CD46) acts as a cellular receptor for measles virus.. J Virol.

[6] Buckland R, Wild TF (1997). Is CD46 the cellular receptor for measles virus?. Virus Res.

[7] Tatsuo H, Ono N, Tanaka K, Yanagi Y (2000). SLAM (CDw150) is a cellular receptor for measles virus.. Nature.

[8] Yanagi Y, Takeda M, Ohno S, Seki F (2006). Measles virus receptors and tropism.. Jpn J Infect Dis.

[9] Leonard VHJ, Sinn PL, Hodge G, Miest T, Devaux P (2008). Measles virus blind to its epithelial cell receptor remains virulent in rhesus monkeys but cannot cross the airway epithelium and is not shed.. J Clin Invest.

[10] Tahara M, Takeda M, Shirogane Y, Hashiguchi T, Ohno S (2008). Measles virus infects both polarized epithelial and immune cells using distinctive receptor-binding sites on its hemagglutinin.. J Virol.

[11] Takeda M, Tahara M, Hashiguchi T, Sato TA, Jinnouchi F (2007). A human lung carcinoma cell line supports efficient measles virus growth and syncytium formation via SLAM- and CD46-independent mechanism.. J Virol.

[12] Takeda M, Takeuchi K, Miyajima N, Kobune F, Ami Y (2000). Recovery of pathogenic measles virus from cloned cDNA.. J Virol.

[13] Hashimoto K, Ono N, Tatsuo H, Minagawa H, Takeda M (2002). SLAM (CD150)-independent measles virus entry as revealed by recombinant virus expressing green fluorescent protein.. J Virol.

[14] De Swart RL, Ludlow M, De Witte L, Yanagi Y, Van Amerongen G (2007). Predominant infection of CD150+ lymphocytes and dendritic cells during measles virus infection of macaques.. PLoS Pathog.

[15] Leonard VH, Hodge G, Reyes-del VJ, McChesney MB, Cattaneo R (2010). Measles virus selectively blind to signaling lymphocytic activation molecule (SLAM; CD150) is attenuated and induces strong adaptive immune responses in rhesus monkeys.. J Virol.

[16] Ludlow M, Rennick L, Sarlang S, Skibinski G, McQuaid S (2010). Wild-type measles virus infection of primary epithelial cells occurs via the basolateral surface without syncytium formation or release of infectious virus.. J Gen Virol.

[17] Shirogane Y, Takeda M, Tahara M, Ikegame S, Nakamura T (2010). Epithelial-mesenchymal transition abolishes the susceptibility of polarized epithelial cell lines to measles virus.. J Biol Chem.

[18] Von Messling V, Svitek N, Cattaneo R (2006). Receptor (SLAM [CD150]) recognition and the V protein sustain swift lymphocyte-based invasion of mucosal tissue and lymphatic organs by a morbillivirus.. J Virol.

[19] De Witte L, Abt M, Schneider-Schaulies S, van Kooyk Y, Geijtenbeek TBH (2006). Measles virus targets DC-SIGN to enhance dendritic cell infection.. J Virol.

[20] De Witte L, De Vries RD, Van der Vlist M, Yüksel S, Litjens M (2008). DC-SIGN and CD150 have distinct roles in transmission of measles virus from dendritic cells to T-lymphocytes.. PLoS Pathog.

[21] El Mubarak HS, Van de Bildt MWG, Mustafa OA, Vos HW, Mukhtar MM (2000). Serological and virological characterization of clinically diagnosed cases of measles in suburban Khartoum.. J Clin Microbiol.

[22] El Mubarak HS, Van de Bildt MWG, Mustafa OA, Vos HW, Mukhtar MM (2002). Genetic characterisation of wild type measles viruses circulating in suburban Khartoum, 1997–2000.. J Gen Virol.

[23] Ibrahim SA, Mustafa OM, Mukhtar MM, Saleh IA, El Mubarak HS (2002). Measles in suburban Khartoum: an epidemiological and clinical study.. Trop Med Int Health.

[24] El Mubarak HS, Yüksel S, Van Amerongen G, Mulder PGH, Mukhtar MM (2007). Infection of cynomolgus macaques (Macaca fascicularis) and rhesus macaques (Macaca mulatta) with different wild-type measles viruses.. J Gen Virol.

[25] De Vries RD, Lemon K, Ludlow M, McQuaid S, Yuksel S (2010). In vivo tropism of attenuated and pathogenic measles virus expressing green fluorescent protein in macaques.. J Virol.

[26] Von Messling V, Milosevic D, Cattaneo R (2004). Tropism illuminated: lymphocyte-based pathways blazed by lethal morbillivirus through the host immune system.. Proc Natl Acad Sci USA.

[27] Kawamata N, Xu B, Nishijima H, Aoyama K, Kusumoto M (2009). Expression of endothelia and lymphocyte adhesion molecules in bronchus-associated lymphoid tissue (BALT) in adult human lung.. Respir Res.

[28] Ferreira CS, Frenzke M, Leonard VH, Welstead GG, Richardson CD (2010). Measles virus infection of alveolar macrophages and dendritic cells precedes spread to lymphatic organs in transgenic mice expressing human signaling lymphocytic activation molecule (SLAM, CD150).. J Virol.

[29] Sternberg S (1997). Histology for pathologists.

[30] Pabst R, Tschernig T (2010). Bronchus-associated lymphoid tissue: an entry site for antigens for successful mucosal vaccinations?. Am J Respir Cell Mol Biol.

[31] Toyoshima M, Chida K, Sato A (2000). Antigen uptake and subsequent cell kinetics in bronchus-associated lymphoid tissue.. Respirology.

[32] Teitelbaum R, Schubert W, Gunther L, Kress Y, Macaluso F (1999). The M cell as a portal of entry to the lung for the bacterial pathogen Mycobacterium tuberculosis.. Immunity.

[33] Morin MJ, Warner A, Fields BN (1994). A pathway for entry of reoviruses into the host through M cells of the respiratory tract.. J Exp Med.

[34] Lemon K, Rima BK, McQuaid S, Allen IV, Duprex WP (2007). The F gene of rodent brain-adapted mumps virus is a major determinant of neurovirulence.. J Virol.

[35] Dubus JC, Vecellio L, De Monte M, Fink JB, Grimbert D (2005). Aerosol deposition in neonatal ventilation.. Pediatr Res.

[36] El Mubarak HS, De Swart RL, Osterhaus ADME, Schutten M (2005). Development of a semi-quantitative real-time RT-PCR for the detection of measles virus.. J Clin Virol.

