# Supplementary figures and images for: Early Target Cells of Measles Virus after Aerosol Infection of Non-Human Primates
Source: PLoS Pathog. 2011 Jan 27;7(1):e1001263. doi: 10.1371/journal.ppat.1001263 (PMC3029373; doi:10.1371/journal.ppat.1001263)

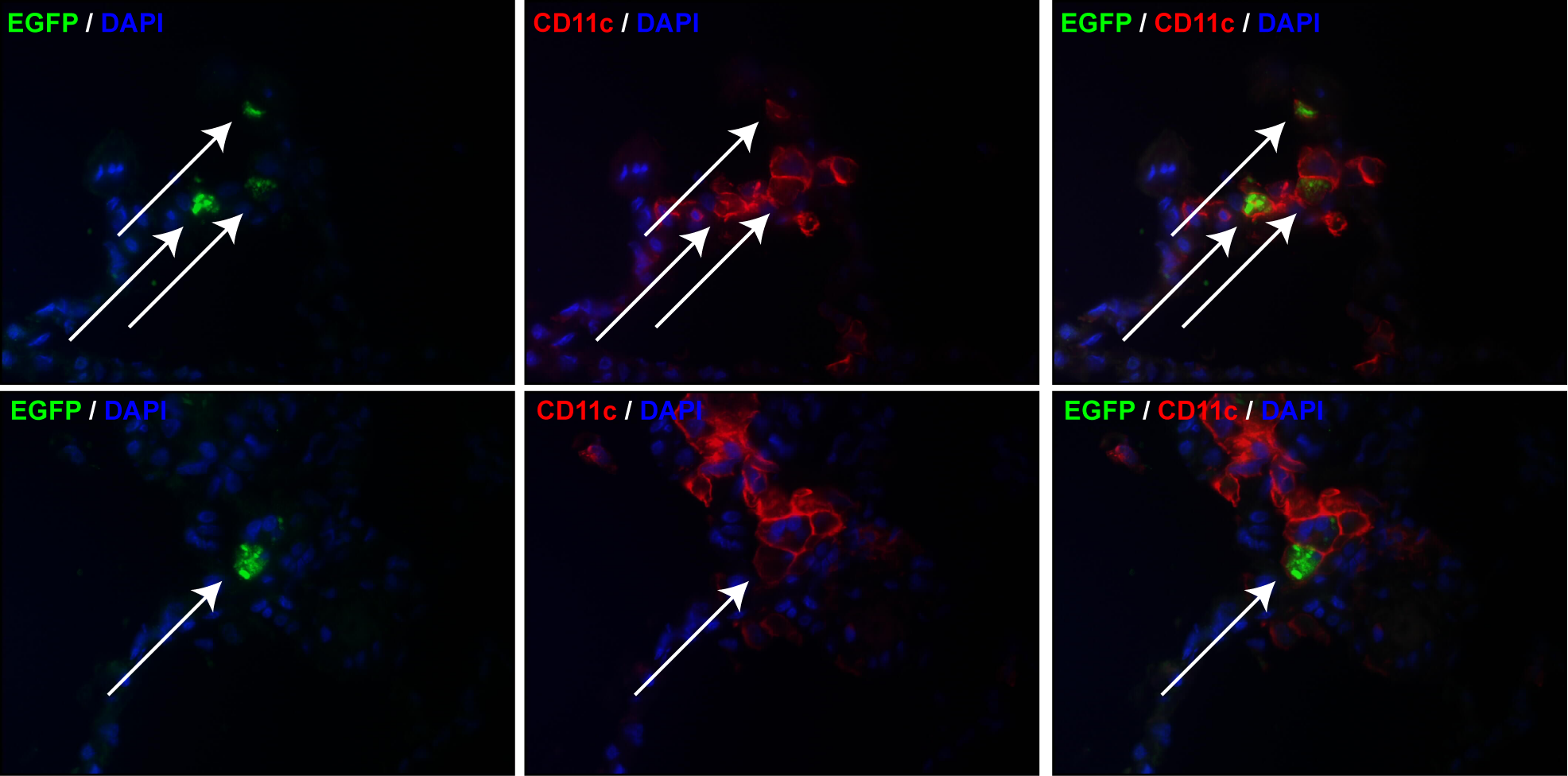

Supplement: Figure S1 — CD11c+ DC and macrophages targeted in the lung at 4 and 5 d.p.i. At 4 and 5 d.p.i. the CD11c+ DC or macrophage population was the major cell type in the lung in which MV replicates. Dual labelling for EGFP (green) and CD11c (red), DAPI was used to counter stain nuclei in blue. Left panels show EGFP alone (green), centre panels show CD11c alone (red), right panels show overlay of EGFP and CD11c. The two rows are two representative examples of double positive cells as indicated by arrows. (2.21 MB TIF) [file ppat.1001263.s001.tif]

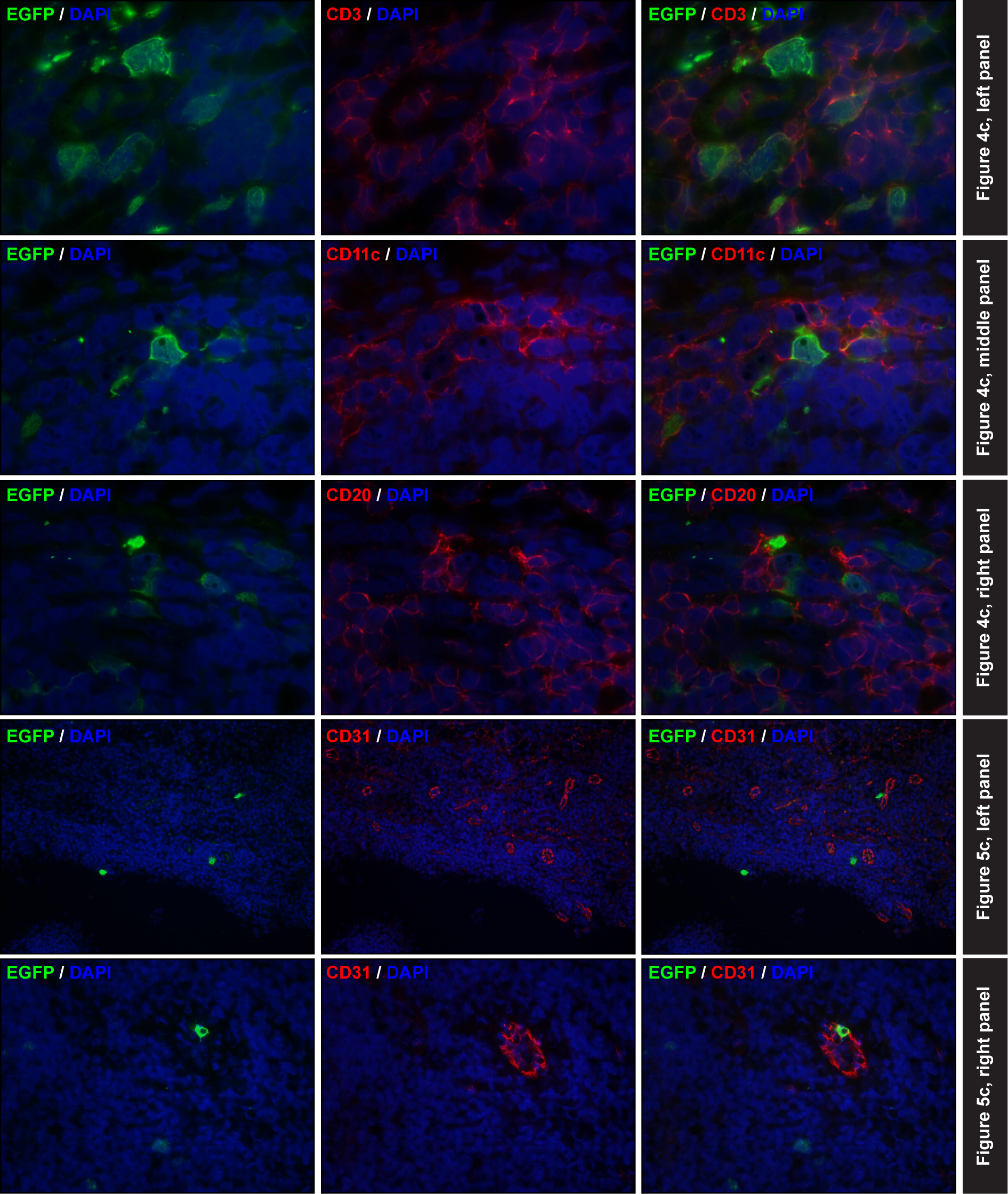

Supplement: Figure S2 — Single color images for figure 4C and 5C. (9.75 MB TIF) [file ppat.1001263.s002.tif]
