# Supplementary material for: Early Target Cells of Measles Virus after Aerosol Infection of Non-Human Primates
Source: PLoS Pathog. 2011 Jan 27;7(1):e1001263. doi: 10.1371/journal.ppat.1001263 (PMC3029373; doi:10.1371/journal.ppat.1001263)
Supplement: Figure S3 — On-line immunohistochemical and H&E pathology scans. Four pathology slides were scanned and digitized at a high resolution and annotated. (0.06 MB PDF) [file ppat.1001263.s003.pdf]

Supporting Figure 3: on-line immunohistochemical and H&E pathology scans. Four pathology slides were scanned and digitized at a high resolution. Areas of interest are annotated in the on-line software program. Sections (A) and (B) are consecutive slides from the lungs of an animal euthanized 3 d.p.i. These sections correspond to figure 4 in the manuscript. Section (A) is stained with a polyclonal rabbit anti-EGFP antibody, counterstained with haematoxilin. Section (B) shows haematoxilin & eosin staining. At this time-point only 2 foci of $EGFP^+$ cells in BALT structures were identified in the lungs of 1 out of 3 animals.

(A) 3 d.p.i. lung section – EGFP
http://www.pathxl.com/iscope/viewer.aspx?resid=28360

(B) 3 d.p.i. lung section - H&E
http://www.pathxl.com/iscope/viewer.aspx?resid=28362

Sections (C) and (D) are consecutive slides from the lungs of an animal euthanized 4 d.p.i. Section (C) is stained with a polyclonal rabbit anti-EGFP antibody, counterstained with haematoxilin. Section (D) shows haematoxilin & eosin staining. At this time-point multiple foci of $EGFP^+$ cells in BALT structures were identified in all animals.

(C) 4 d.p.i. lung section – EGFP
http://www.pathxl.com/iscope/viewer.aspx?resid=28364

(D) 4 d.p.i. lung section - H&E.
http://www.pathxl.com/iscope/viewer.aspx?resid=28365

Select the hyperlinks to access the data and obtain annotated descriptions of the pathological assessments for each slide. Under the submenu ‘image’, you have the option ‘sharpen image’ to obtain a sharper image. In the lower middle part of the screen you can adjust the zoom settings to zoom in on areas of interest.

Visualizing the small areas of infected AM and BALT in the context of an entire 7 µM tissue section illustrates the power of EGFP-driven targeted pathology. Such pathological assessments would be impossible to make in the absence of being able to triage the large majority of virus-negative lung samples during early time point necropsies.
